# MHC Class I Chain-Related Gene A Polymorphisms and Linkage Disequilibrium with HLA-B and HLA-C Alleles in Ocular Toxoplasmosis

**DOI:** 10.1371/journal.pone.0144534

**Published:** 2015-12-16

**Authors:** Christiane Maria Ayo, Ana Vitória da Silveira Camargo, Fábio Batista Frederico, Rubens Camargo Siqueira, Mariana Previato, Fernando Henrique Antunes Murata, Aparecida Perpétuo Silveira-Carvalho, Amanda Pires Barbosa, Cinara de Cássia Brandão de Mattos, Luiz Carlos de Mattos

**Affiliations:** 1 Immunogenetics Laboratory, Molecular Biology Department, Faculdade de Medicina de São José do Rio Preto de São José do Rio Preto, SP, Brazil; 2 Ophthalmology Outpatient Clinic, Hospital de Base de São José do Rio Preto, Fundação Faculdade Regional de Medicina de São José do Rio Preto, SP, Brazil; 3 FAMERP Toxoplasma Research Group, Fundação Faculdade Regional de Medicina de São José do Rio Preto, São José do Rio Preto, SP, Brazil; Institut national de la santé et de la recherche médicale—Institut Cochin, FRANCE

## Abstract

This study investigated whether polymorphisms of the *MICA* (major histocompatibility complex class I chain-related gene A) gene are associated with eye lesions due to *Toxoplasma gondii* infection in a group of immunocompetent patients from southeastern Brazil. The study enrolled 297 patients with serological diagnosis of toxoplasmosis. Participants were classified into two distinct groups after conducting fundoscopic exams according to the presence (n = 148) or absence (n = 149) of ocular scars/lesions due to toxoplasmosis. The group of patients with scars/lesions was further subdivided into two groups according to the type of the ocular manifestation observed: primary (n = 120) or recurrent (n = 28). Genotyping of the MICA and HLA alleles was performed by the polymerase chain reaction-sequence specific oligonucleotide technique (PCR-SSO; One Lambda®) and the MICA-129 polymorphism (rs1051792) was identified by nested polymerase chain reaction (PCR-RFLP). Significant associations involving MICA polymorphisms were not found. Although the MICA*002~HLA-B*35 haplotype was associated with increased risk of developing ocular toxoplasmosis (*P*-value = 0.04; OR = 2.20; 95% CI = 1.05–4.60), and the MICA*008~HLA-C*07 haplotype was associated with protection against the development of manifestations of ocular toxoplasmosis (*P*-value = 0.009; OR: 0.44; 95% CI: 0.22–0.76), these associations were not statistically significant after adjusting for multiple comparisons. MICA polymorphisms do not appear to influence the development of ocular lesions in patients diagnosed with toxoplasmosis in this study population.

## Introduction

Ocular toxoplasmosis, characterized by intraocular inflammation, is the most common clinical manifestation of toxoplasmosis, the infectious disease caused by *Toxoplasma gondii* [1]. Lesions originate both from congenital infection and from infections acquired after birth [2,3]. The lesions can affect the macula and other layers of the retina and the choroid, resulting in retinochoroiditis, the most frequent cause of posterior uveitis in immunocompetent patients [1]. Ocular manifestations can have an early or late onset, with primary or recurrent clinical manifestations [4] and present different degrees of ocular involvement that vary according to the immune status of the individual [1,5] and different *T*. *gondii* strains [6–8]. Whether the ocular manifestation resulting from infection by *T*. *gondii* is attributable to host or parasite genetic factors or differences in exposure rate remains uncertain [9].

The *MICA* gene is located on chromosome 6 in the region of the class I major histocompatibility complex gene (*MHC*) close to the *HLA-B* and *HLA-C* gene loci [10]. Under stress the *MICA* gene encodes a cell surface protein that is a ligand for NKG2D, an activating receptor of Tγδ lymphocytes, CD8^+^ Tαβ lymphocytes and natural killer cells (NK) [11,12]. The MICA alleles can be categorized as strong (MICA-129 met) or weak ligands (MICA-129 val) of the NKG2D receptor based on the MICA 129 polymorphism (rs1051792). Corresponding to the 129 amino acid of the protein, this polymorphism alters a single amino acid with the substitution of methionine to valine (A>G) at position 454 of the third exon of the *MICA* gene and most likely alters the activation of these cells [13].

Besides the involvement of both CD8^+^ T lymphocytes and NK cells, the immune response to *T*. *gondii* infection is also characterised by a strong T helper-1 (Th-1) response orchestrated by CD4^+^ T cells and dominated by the production of proinflammatory mediators. However, while the Th-1 response prevents parasite replication, the strong Th-1 response may also cause immune-mediated tissue damage contributing to the severity of ocular toxoplasmosis. More recently, Th-17 cells, characterised by the production of interleukin-17 (IL-17), a potent inducer of inflammation, have been identified as key contributors to immunopathological responses in ocular toxoplasmosis [14–16].

MICA polymorphisms are possibly associated with the susceptibility or progression of several infectious diseases such as dengue fever [17], leprosy [18], tuberculosis [19], schistosomiasis [20], and Chagas disease [21,22], among others. Furthermore, the expression of MICA in inflamed tissues or in autoimmune diseases, in particular the MICA-129 polymorphism, would contribute to the immunopathology of these diseases [22–27]. However, the role of the MICA alleles and the effect of the MICA-129 functional polymorphism in ocular toxoplasmosis remain unknown and there is no data on the expression of MICA in ocular tissue affected by *T*. *gondii*.

This study investigated whether the MICA alleles and the 129 polymorphism in exon 3 of the *MICA* gene are associated with the development of eye lesions resulting from *T*. *gondii* infection in a group of immunocompetent patients from southeastern Brazil.

## Materials and Methods

### Ethics information

This study was approved by the Research Ethics Committee of the School of Medicine in São José do Rio Preto (#1980/2009) and all individuals who agreed to participate in this research were informed about the nature of the study and signed informed consent forms.

### Sample selection

A total of 297 unrelated patients were selected from those seeking ophthalmological treatment in the Retinopathy Outpatient Service of Hospital de Base of the School of Medicine in São José do Rio Preto and Medical Outpatient Clinic (AME) in São José do Rio Preto. All patients selected for this study had positive serology for *T*. *gondii*. *A*nti*-T*. *gondii* antibodies were detected by immunosorbent assay (ELISA) according to the manufacturer's instructions (ETI-TOXOK-M reverse PLUS; DiaSorin S.p.A. Italy and ETI-TOXO-G PLUS; DiaSorin S.p.A. Italy). Of all the patients included in this study, only five of those with ocular scars/lesions due to toxoplasmosis showed positive serology for both IgM and IgG anti*-T*. *gondii* antibodies. The remaining patients only had positive serology for IgG anti*-T*. *gondii* antibodies.

The clinical evaluation of patients was conducted by two experienced physicians using an indirect binocular ophthalmoscope (Binocular Ophthalmoscope ID10, Topcon Corporation, USA) as previously described [28]. Subsequently, patients were classified into two distinct groups according to the presence of ocular scars/lesions due to toxoplasmosis (n = 148; 79 men and 69 women; mean age: 42.3 ± 20.6 years) or to the presence of ocular diseases other than toxoplasmosis (n = 149; 73 men and 76 women; mean age: 57.7 ± 16.9 years) such as cataracts (17.5%), pterygium (4.0%), age-related macular degeneration (23.0%), glaucoma (6.7%), retinal detachment (16.1%), optic neuropathy (3.4%), macular edema (4.6%), macular atrophy (2.6%), diabetic retinopathy (8.7%) and other ocular diseases (13.4%). The group of patients with scars/lesions due to toxoplasmosis was further subdivided into two groups according to the type of ocular manifestation observed during a follow up period of at least two years: primary manifestations (n = 120; 65 men and 55 women; mean age: 44.9 ± 20.9 years) and recurrent manifestations characterised by the presence of satellite lesions (n = 28; 14 men and 14 women; mean age: 31.8 ± 30.5 years) (Table 1) [29].

**Table 1:** General characteristics of patients with and without ocular toxoplasmosis and its manifestation as primary or recurrent

Characteristic	Patients without ocular toxoplasmosis (n = 149)	Patients with ocular toxoplasmosis (n = 148)	Patients with primary manifestation (n = 120)	Patients with recurrent manifestation (n = 28)
				
Age (Mean ± SD)	57.7±16.9^a^ ^,^ ^b^ ^,^ ^c^	42.3±20.6^a^	44.9±20.9^b^ ^,^ ^d^	31.8±30.5^c^ ^,^ ^d^
Median	60	37	46	30
Gender (%)				
Female	76 (51.0%)	69 (46.6%)	55 (45.8%)	14 (50.0%)
Male	73 (49.0%)	79 (53.4%)	65 (54.2%)	14 (50.0%)

t = Student t test.

^a^ *P-value<*0.0001 t = 7.00 (Patients without ocular toxoplasmosis vs. Patients with ocular toxoplasmosis)

^b^ *P-value<*0.0001 t = 5.48 (Patients without ocular toxoplasmosis vs. Patients with primary manifestation)

^c^ *P-value<*0.0001 t = 7.51 (Patients without ocular toxoplasmosis vs. Patients with recurrent manifestation)

^d^ *P-value* = 0.002 t = 3.12 (Patients with primary manifestation vs. Patients with recurrent manifestation)

All patients underwent detailed eye examinations including visual acuity (logMAR Early Treatment Diabetic Retinopathy Study [ETDRS] chart) with best correction according to the ETDRS standards [30], measurement of intraocular pressure by Goldmann applanation tonometry, biomicroscopy using a slit lamp, and stereoscopic biomicroscopy performed using a 78-diopter lens (Volk) and classified according to the ETDRS criteria.

**Fig 1:**
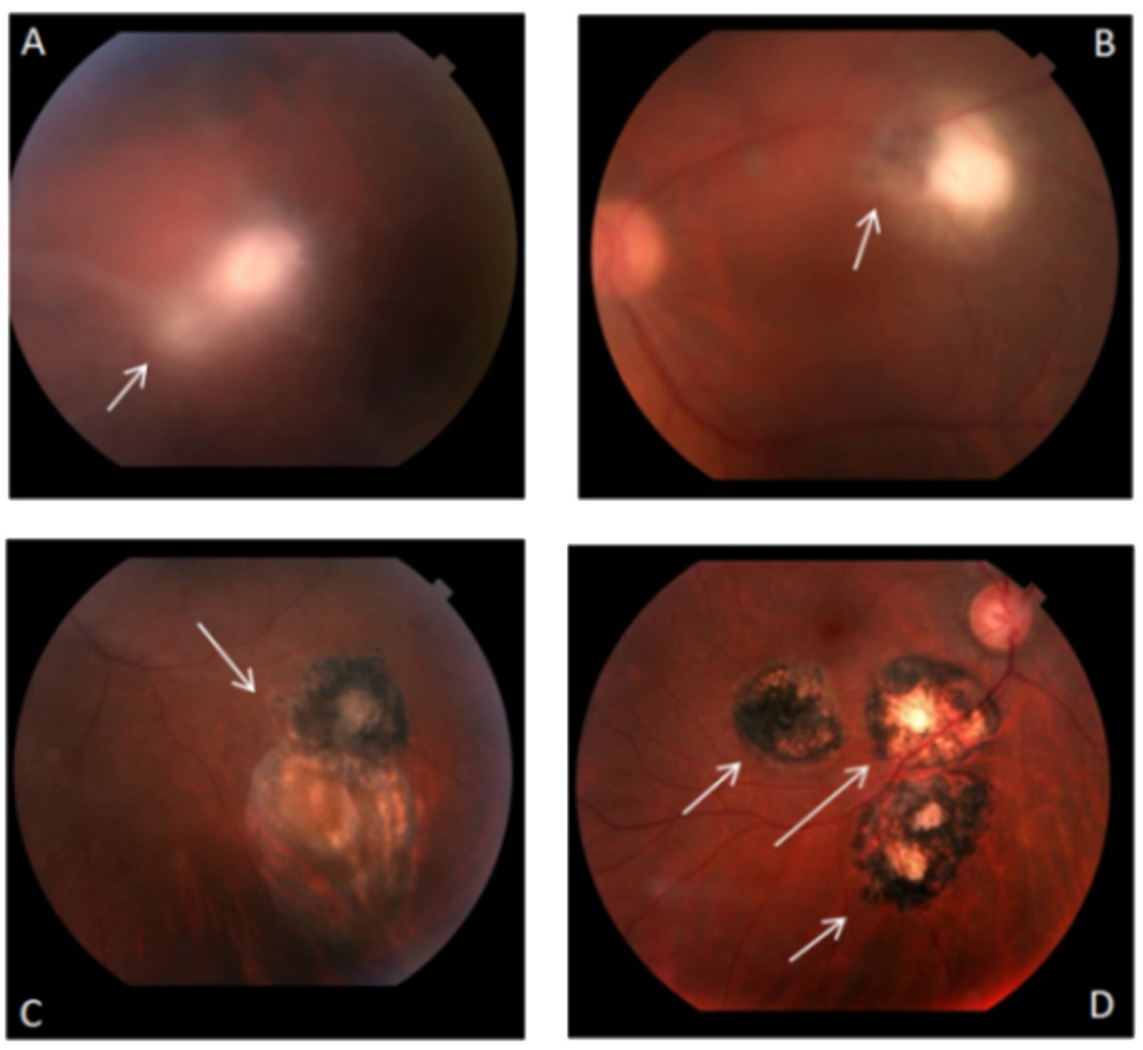
Colour retinography showing the various stages of eye lesions caused by *Toxoplasma gondii* infection in Brazilian patients. In (A) the arrow indicates the region with an acute exudative chorioretinal lesion ("lighthouse in the fog") and cloudy vitreous. In (B) the arrow indicates a chorioretinal lesions in the healing process—the patient had good clinical response to treatment and scar edges in definition. In (C) the arrow indicates presentation of an old chorioretinal scar and an old chorioretinal satellite lesion with pigment mobilization. In (D) chorioretinal scaring with well-defined edges indicated by the arrows with visualization of the sclera.

Colour fundus photographs and fluorescent photographs were taken using a digital retinal camera (TRC-50DX, Topcon Medical Systems) to document the macula region and optic nerve. Areas of progressive hyperfluorescence (leakage), staining and transmitted hyperfluorescence (window effect) were investigated by fluorescein angiography. Progressive hyperfluorescence with late leakage was considered a sign of lesion activity. Fig 1 shows the different stages of eye lesions caused by *T*. *gondii* infection by colour retinography.

Although the patients in this study were of European descent, mixed African and European descent, and African descent, they were grouped as a population of mixed ethnicity due to high miscegenation of the Brazilian population [31]. The risk of population stratification bias between patients with scars/lesions and patients without ocular manifestations was minimized by matching the ethnic background, gender and geographic area of residence. These data were carefully checked to select groups.

### DNA extraction and MICA, HLA-B, and HLA-C genotyping

Genomic DNA was attained from peripheral blood using a commercial kit for silica column extraction (QIAamp® DNA Blood Mini Kit, QIAGEN, the Netherlands) following the manufacturer's instructions. The genotyping of the MICA, HLA-B and HLA-C alleles was performed using the polymerase chain reaction-sequence specific oligonucleotide (PCR-SSO) technique with the rSSO Luminex® genotyping kit (One Lambda, Canoga Park, CA, USA). This technique first targets PCR-amplified DNA using specific biotinylated primers and subsequently the amplified product is hybridized by complementary DNA probes conjugated to fluorescently coded microspheres, with detection using R-Phycoerythrin-conjugated Streptavidin (SAPE). Hybridization was verified by flow cytometry (LABScanTM 100 flowanalyzer) and data were interpreted using computer software (HLA Fusion, version 3.4, One Lambda®).

The 129 A>G (rs1051792) polymorphism of the *MICA* gene was identified by nested polymerase chain reaction (PCR-RFLP) using a technique adapted from Amroun et al. [23]. In this technique, the *MICA* gene-specific amplicon was used as a template in a second round amplification of its exon 3. The primer pair used in the first reaction was 5' CGT TCT TGT CCC TTT GCC CGT GTG C 3' and 5' GAT GCT GCC CCC ATT CCC TTC CCA AA 3' with an initial denaturation at 95°C for 5 min, followed by 40 cycles of 95°C for 45 s, 65°C for 45 s, 72°C for 45 s and a final extension at 72°C for 5 min. The primer pair used in the second reaction was 5' GGG TCT GTG AGA TCC ATG A 3' and 5' TGA GCT CTG GAG GAC TGG GGT A 3' with an initial denaturation at 95°C for 5 min, followed by 25 cycles of 95°C for 45 s, 61°C for 45 s, 72°C for 45 s and a final extension at 72°C for 5 min. The MICA-129 val allele was identified by the presence of a restriction site for the RsaI enzyme (FastDigest ™, Thermo Scientific, USA) created by a mismatch introduced into the nonsense primer. For reasons of clarity and to follow the published nomenclature, the alleles will be designated here as MICA-129 met and MICA-129 val.

### Statistical analysis

Genotype frequencies were obtained by direct counting, while the ARLEQUIN software (version 3.11; http://cmpg.unibe.ch/software/arlequin3) was used to calculate the allele and haplotype frequencies. The haplotype frequency was estimated by the expectation-maximization algorithm method [32], which allows an estimation of random haplotype frequencies based on the allele frequencies of the sample. Relative linkage disequilibrium (Δ') was calculated according to the Imanishi method [33], and the Hardy-Weinberg equilibrium was verified according to the method described by Guo & Thompson [34].

Comparisons of allele, haplotype and genotype frequencies between groups of patients were attained using the chi-square test with Yates’ correction or Fisher’s exact test. Odds ratio (OR) with a 95% confidence interval (95% CI) was also calculated to evaluate the risk association. The mean ages were compared using the t-test. Differences, considered statistically significant for *P*-values ≤0.05, were corrected by the Bonferroni inequality method for multiple comparisons (*Pc*). Statistical analyses were performed using the GraphPad Instat software (version 3.06).

## Results

### General characteristics of patients with and without ocular manifestations of toxoplasmosis

The general characteristics of the study participants are shown in Table 1. The group of patients without ocular toxoplasmosis presented a significantly higher mean age compared with the group of patients with ocular toxoplasmosis (*P*-value <0.0001; t = 7.00), with the subgroup of patients with primary manifestations of ocular toxoplasmosis (*P*-value <0.0001; t = 5.48) and with the subgroup of patients with the recurrent form of the disease (*P*-value <0.0001; t = 7.51). Differences in age were also observed between the subgroups of patients: those with the primary manifestation of the disease had a higher mean age than those who had recurrent manifestations (*P*-value = 0.002; t = 3.12).

### Frequency of MICA alleles in patients with and without ocular manifestations of toxoplasmosis

Table 2 shows the distribution of the MICA alleles. Sixteen alleles were identified in the sample of patients without ocular toxoplasmosis and 20 in patients with ocular toxoplasmosis. The most common alleles in both groups were MICA*008, MICA*002, MICA*004 and MICA*009 totalling 63.5% and 64.8% of the possible alleles in patients with and without ocular toxoplasmosis, respectively. The MICA*030, MICA*041, MICA*044 and MICA*068 alleles were present only in patients with ocular manifestations of toxoplasmosis, with only one individual having each allele (0.3%). No significant differences were found in the distribution of MICA alleles between the groups of patients with and without ocular toxoplasmosis or between those with primary or recurrent clinical manifestations of the disease, so that the distribution of these alleles is in Hardy-Weinberg equilibrium in the study population.

**Table 2:** Distribution of MICA alleles in patients with and without ocular toxoplasmosis and its manifestation as primary or recurrent

MICA alleles	Patients without ocular toxoplasmosis (n = 149)	Patients with ocular toxoplasmosis (n = 148)	Patients with primary manifestation (n = 120)	Patients with recurrent manifestation (n = 28)
	N (%)	N (%)	N (%)	N (%)
*001	7 (4.7)	6 (4.1)	6 (2.5)	0 (0.0)
*002	50 (33.6)	51 (34.5)	37 (15.5)	14 (25.0)
*004	40 (26.8)	37 (12.5)	32 (13.3)	5 (8.9)
*006	1 (0.7)	1 (0.7)	1 (0.4)	0 (0.0)
*007	11 (7.4)	14 (9.5)	11 (4.6)	3 (5.4)
*008	65 (43.6)	59 (39.9)	48 (20.0)	11 (19.6)
*009	38 (25.5)	41 (27.7)	37 (15.4)	4 (7.1)
*010	15 (10.1)	15 (10.1)	11 (4.6)	4 (7.1)
*011	11 (7.4)	17 (11.5)	15 (6.3)	2 (3.6)
*012	4 (2.7)	3 (2.0)	3 (1.3)	0 (0.0)
*015	5 (3.4)	4 (2.7)	3 (1.3)	1 (1.8)
*016	16 (10.7)	10 (6.8)	8 (3.3)	2 (3.6)
*017	5 (3.4)	9 (6.1)	7 (2.9)	2 (3.6)
*018	16 (10.7)	10 (6.8)	8 (3.3)	2 (3.6)
*019	7 (4.7)	6 (4.1)	3 (1.3)	3 (5.4)
*027	8 (5.4)	9 (6.1)	5 (2.1)	3 (5.4)
*030	0 (0.0)	1 (0.7)	1 (0.4)	0 (0.0)
*041	0 (0.0)	1 (0.7)	1 (0.4)	0 (0.0)
*044	0 (0.0)	1 (0.7)	1 (0.4)	0 (0.0)
*068	0 (0.0)	1 (0.7)	1 (0.4)	0 (0.0)

N: number of alleles

### Frequency of MICA-129 genotypes and alleles in patients with and without ocular manifestations of toxoplasmosis

There were no associations of genotypes or alleles of the MICA-129 polymorphism between the groups of patients with and without ocular toxoplasmosis or between the subgroups of patients with primary or recurrent ocular manifestations of the disease. The MICA-129 val allele and the heterozygous MICA-129 met/val genotypes were the most common in all groups (Table 3).

**Table 3:** Genotype and allele frequencies of the MICA-129 polymorphism (rs1051792) in patients with and without ocular toxoplasmosis and its manifestation as primary or recurrent

MICA-129 polymorphism	Patients without ocular toxoplasmosis (n = 149)	Patients with ocular toxoplasmosis (n = 148)	Patients with primary manifestation (n = 120)	Patients with recurrent manifestation (n = 28)	*P*-value
			
Genotypes	n (%)	n (%)	n (%)	n (%)	
met/met	22 (14.8)	22 (14.9)	18 (15.0)	4 (14.3)	ns
met/val	64 (43.0)	73 (49.3)	57 (47.5)	16 (57.1)	ns
val/val	63 (42.3)	53 (35.8)	45 (37.5)	8 (28.6)	ns
Alleles	N (%)	N (%)	N (%)	N (%)	
met	108 (36.2)	117 (39.5)	93 (38.8)	24 (42.9)	ns
val	190 (63.8)	179 (60.5)	142 (59.2)	32 (57.1)	ns

N: number of alleles; ns: non-significant (when *P-*value >0.05)

### Frequency of MICA~HLA-B and MICA~HLA-C haplotypes in patients with and without ocular manifestations of toxoplasmosis

The most common MICA~HLA haplotypes are shown in Table 4. The MICA*002~HLA-B*35 haplotype was associated with increased risk of developing ocular toxoplasmosis (*P*-value = 0.04; OR: 2.20; 95% CI: 1.08–4.93), while the MICA*008~HLA-C*07 haplotype was associated with protection against the development of manifestations of ocular toxoplasmosis (*P*-value = 0.009; OR: 0:44; 95% CI: 0.22–0.76). However, the significance of these associations was not statistically significant after correcting for multiple comparisons. There was no significant difference on comparing the MICA~HLA-B and MICA~HLA-C haplotypes between patients with primary manifestations and those with recurrent manifestations of ocular toxoplasmosis.

**Table 4:** Haplotype frequencies of MICA, HLA-B and HLA-C in patients with and without ocular toxoplasmosis and its manifestation as primary or recurrent

Haplotypes		Patients without ocular toxoplasmosis (n = 149)		Patients with ocular toxoplasmosis (n = 148)		Patients with primary manifestation (n = 120)		Patients with recurrent manifestation (n = 28)					
MICA	HLA	n	(%)	n	(%)	n	(%)	n	(%)	*P*-value	*Pc*	OR	IC (95%)
*002	B*35	11	3.6	23	7.8	17	7.0	6	10.7	0.04^a^ ^vs.^ ^b^	ns	2.20	1.05–4.60
*002	B*53	14	4.7	6	2.0	4	1.7	2	3.6	Ns			
*004	B*44	26	8.7	14	4.7	11	4.6	3	5.3	Ns			
*008	B*07	19	6.3	10	3.3	8	3.3	2	3.5	Ns			
*009	B*51	16	5.3	21	7.0	17	7.0	3	5.3	Ns			
*002	C*04	25	8.3	30	9.8	22	9.2	8	14.3	Ns			
*004	C*16	18	6.0	12	4.0	10	4.1	2	3.5	Ns			
*008	C*07	36	12.0	17	5.7	16	6.6	1	1.7	0.009^a^ ^vs.^ ^b^	ns	0.44	0.22–0.76
*009	C*06	12	3.9	7	2.4	7	2.9	0	0.0	ns			
*016	C*04	13	4.4	11	3.4	9	3.1	2	3.5	ns			

ns: non-significant (when *P-*value >0.05)

^a^: Patients without ocular toxoplasmosis

^b^: Patients with ocular toxoplasmosis

## Discussion

Although the importance of NK cells and CD8^+^ T lymphocytes to the immune response of individuals infected by *T*. *gondii* [35,36] and the role of MICA alleles in the activation of these cells has been established [8,9], to the best of our knowledge, this is the first study that addresses the role of MICA alleles and the MICA-129 polymorphism (rs1051792) in the immunopathogenesis of toxoplasmosis. Several factors related to both the host and the parasite have been suggested as possible causes of the initial manifestation and the recurrence of ocular toxoplasmosis, but none is widely accepted [5,6,37,38].

The average ages of the group and subgroups of patients with ocular toxoplasmosis are less than the average age of patients without ocular toxoplasmosis. Moreover, patients presenting recurrent ocular manifestations have a lower mean age than those with primary disease. Several studies have reported the importance of age in the clinical course of ocular toxoplasmosis with most of them showing that the disease most often affects patients from the second to fourth decades of life [29,39–41]. Moreover, the risk of recurrence is higher in the year following the first episode than in following years [42]. A common feature of these studies (including this study) is that the patients with ocular toxoplasmosis were relatively young. Indeed, other eye diseases, those without ocular scars/lesions due to toxoplasmosis, are prevalent in older patients [43], although ocular toxoplasmosis may develop at any stage of life [4]. The majority of cases of ocular involvement due to toxoplasmosis are considered postnatally acquired infections [1].

No distinction was made between congenital and acquired disease in the analysis of the characteristics of eye injuries. During infection, a pregnant woman presents a temporary parasitaemia, which, can cause focal lesions in the placenta and infect the fetus, with varying severity of damage, depending on the virulence of the parasite strain, the immune response of the mother and the gestational period [44]. Reactivation of chronic *T*. *gondii* infection and consequent disease is common in congenitally infected individuals and some studies report that ocular disease is the most common manifestation of congenital toxoplasmosis [45–48]. In addition to the presence of clinically detectable ocular lesions at birth, new lesions typically appear late in children who receive treatment or not [49,50], although the recurrence rates of congenital and acquired ocular toxoplasmosis appear to be similar [29].

It has been postulated that recurrence is associated with reactivation of cysts in the retina attributed to immaturity or alterations in host immunity [51]. Both patients with congenital infection and older patients seem to be at higher risk of developing ocular lesions [47]. However, independent studies show that individuals with primary toxoplasmic retinochoroiditis without a pre-existing retinochoroidal lesion were older than those with recurrent ocular toxoplasmosis [29,52]. Furthermore, it is possible that recurrent ocular manifestations result from repeated infections of more than one strain of the parasite [53,54], or they may also be associated with more virulent parasite strains [6,7,55].

The frequency of the MICA alleles in this study population was similar to that found by Marin *et al*. [56] in a healthy population of the state of São Paulo and Ribas *et al*. [57] in a healthy population from the state of Paraná, with the MICA*008, *002, *004 and *009 alleles being the most common. However, the data of this study do not suggest that the extensive allelic polymorphism of the *MICA* gene contributes independently from HLA-B and HLA-C to the appearance of ocular lesions resulting from *T*. *gondii* infection.

Furthermore, comparisons between the MICA alleles revealed differences in their ability to bind to the NKG2D receptor. Sequences whose codon 129 is encoded with methionine express proteins with a 10 to 50 times greater capacity to form a complex with NKG2D than sequences with valine at this position, which possibly affects the activation and modulation of NK and T cells [13]. However, our results showed there were no associations of the genotypes or alleles of the MICA-129 polymorphism between the groups of patients diagnosed with toxoplasmosis (with or without ocular injury) or between the subgroups of patients with manifestations of primary or recurrent disease.

The intraocular immune response is suppressed in normal circumstances thereby decreasing the risk of tissue destruction [58]. Under these conditions, cells in different tissues of the eye constitutively express the Fas ligand (Fas-L), which can promote the deletion of T cells and NK cells in the eye. Moreover, cytokines, such as transforming growth factor-beta (TGF-β), which have immunosuppressive properties, are also present, reducing the expression of MHC class I molecules [59,60], which may affect cytotoxic lymphocyte responses. However, decreased levels of TGF-β were found in ocular fluids of individuals with uveitis [61] and it has been shown that *T*. *gondii* is capable of stimulating the release and modifying the active form of TGF-β thereby facilitating replication of the parasite [62].

The pathogenesis of inflammation in ocular toxoplasmosis remains unclear, but several theories have been proposed in an attempt to explain this process [5]. There is evidence that *T*. *gondii* infection promotes the production of factors, such as interferon-gamma (IFN-γ), that suppress immune privilege which has a crucial role in protecting against infection, as well as being a potent TGF-β antagonist and hyper-regulating the expression of MHC molecules [63,64]. Murine models have shown that the ocular immune response against *T*. *gondii* involves factors similar to responses in other tissues, possibly leading to increased severity of lesions characterized by marked necrosis or inflammation of the retina and the choroid [65–67].

So far it has been shown that MICA alleles participate in the rejection process of solid organ transplants, immune surveillance of tumours and viruses [68] and the progression of several infectious [17–22], inflammatory and autoimmune diseases [23–27]. However, there is no evidence that immunopathogenic mechanisms related to diseases that involve MICA molecules also act on the immunity of ocular tissue affected by *T*. *gondii*, as the nature of the expression of MICA as a response to this parasite infection has not been demonstrated in these tissues yet. It has only been reported that, *in vitro*, the MICA protein has a reduced expression in normal corneal epithelium and that an increase in the expression of this protein results in cytotoxic activity of NK cells and CD8^+^ T cells [69].

Lymphocytes expressing the NKG2D receptor are present in the eye during episodes of inflammation [58,70], and there is evidence that both NK and CD8^+^ T cells are important components of the immune response against *T*. *gondii*. It is known that the expression of MICA molecules increases in response to infections and can trigger cytotoxicity and IFN-γ secretion by cells expressing the NKG2D receptor [71]. This study investigated whether the MICA allele and MICA-129 polymorphism, which affect binding affinity to the NKG2D receptor, are associated with the onset of ocular lesions in patients who are serologically positive for toxoplasmosis; however, no correlation was found.

Another possibility is that some MICA allotypes are intimately linked to other alleles responsible for this association, such as HLA, due to the relatively close physical proximity between their loci. According to Stephens [72], it is common for MICA alleles to be associated with HLA alleles, principally with HLA-B and thus exert a synergistic effect when combined. The association between MICA and ocular toxoplasmosis was observed only when the linkage disequilibrium between the HLA-B and HLA-C loci was analysed. The MICA*002~HLA-B*35 haplotype was associated with increased risk of developing ocular toxoplasmosis, while the MICA*008~HLA-C*07 haplotype was associated with protection against the ocular manifestations of toxoplasmosis. A relatively significant linkage disequilibrium value was observed for the MICA*002~HLA-B*35 haplotype in patients who developed ocular symptoms (Δ' = 0.4305; *P*-value = 0.002), and for the MICA*008~HLA-C*07 haplotype in the group of patients without ocular manifestations of the disease (Δ' = 0.3582; *P*-value = 0.001).

When the alleles that make up the haplotypes listed above were analysed separately, no association was detected in respect to ocular toxoplasmosis or to the primary or recurrent clinical forms of the disease (data not shown for the HLA-B and HLA-C loci), so the possibility that the HLA alleles are mainly responsible for the association can be excluded. On the other hand, we cannot exclude chance as an explanation for the observed associations, as the statistical significance was no longer statistically significant after correcting for multiple comparisons. Moreover, as the haplotype frequencies were obtained from the allele frequencies, it is important to emphasize that they may not be accurate. To evaluate the true haplotype distribution, it is necessary to know the ancestors of the individuals in order to identify inherited haplotypes; this was not possible in the current study.

In conclusion, in this study population, the MICA alleles and MICA functional polymorphism-129 do not seem to influence the development of ocular lesions in patients diagnosed with toxoplasmosis. For a better understanding of the influence of MICA~HLA haplotypes as risk factors for ocular toxoplasmosis we suggest that additional studies should be conducted, in particular involving families. As allelic diversity of the *MICA* gene can differ between populations, according to regional variations, associations involving MICA polymorphisms could result in different clinical and immune phenotypes in patients with ocular toxoplasmosis from less racially mixed populations [16]. Furthermore, it is important to emphasize that the ocular toxoplasmosis diagnostic criteria used in this study were the same as in the clinical practice, injury identified by ophthalmoscopy associated with positive serology for *T*. *gondii*. As no invasive test was performed, this is a presumptive diagnosis when antibodies, antigens and protozoas were not detected in the injury. Furthermore, a histological analysis of the ocular tissue affected by *T*. *gondii* is necessary as well as investigations of the cytotoxicity of NK and CD8^+^ T cells to clarify the expression of MICA molecules and to gain a better understanding of the role of cells expressing the NKG2D receptor in the immunopathogenesis of ocular toxoplasmosis.
